# The epigenome of synovial fibroblasts: an underestimated therapeutic target in rheumatoid arthritis

**DOI:** 10.1186/ar4596

**Published:** 2014-06-26

**Authors:** Mojca Frank-Bertoncelj, Steffen Gay

**Affiliations:** 1 Center of Experimental Rheumatology, University Hospital Zurich, Gloriastrasse 23, Zurich CH-8091, Switzerland

## Abstract

Perturbed epigenetic landscape and deregulated microRNA networks are central to the permanent activation and aggressiveness of synovial fibroblasts in rheumatoid arthritis. Current anti-cytokine therapies, although effectively halting synovitis, cannot reverse the stably activated destructive phenotype of rheumatoid arthritis synovial fibroblasts, offering rather limited protection against ongoing joint destruction in rheumatoid arthritis. Targeting the deregulated epigenome of rheumatoid arthritis synovial fibroblasts is key to developing joint-protective strategies in rheumatoid arthritis. To date, different pathogenic mechanisms have been identified that can profoundly impact the epigenetic derangements in rheumatoid arthritis synovial fibroblasts, including increased consumption of S-adenosylmethionine, a principal methyl donor in DNA methylation reactions, together with deregulation of crucial DNA- and histone-modifying enzymes. Re-establishing globally disturbed DNA methylation patterns in rheumatoid arthritis synovial fibroblasts by supplementing S-adenosylmethionine while preventing its leakage into polyamine cycles may be a promising therapeutic strategy in rheumatoid arthritis and the first epigenetic treatment to target rheumatoid arthritis synovial fibroblasts at the scene of the crime. Given the dynamic nature and reversibility of epigenetic modifications, their involvement in human diseases and recent perspectives on epigenetic therapies in cancer, epigenetic targeting of rheumatoid arthritis synovial fibroblasts should be within future reach.

## 

Rheumatoid arthritis (RA) is characterized by inflammation and progressive destruction of joints, resulting in pain and functional disability. Resident synovial cells, RA synovial fibroblasts (RASF), are major effectors of joint destruction and active contributors to joint inflammation [1]. The aggressive, invasive phenotype of RASF appears early in RA as a consequence of stable cell activation. Several key factors in the pathogenesis of RA, including proinflammatory cytokines, innate immunity and matrix-degradation products, critically amplify activation of RASF [2]; it remains unclear, however, whether they are also indispensable to early cell activation.

The altered epigenome, including DNA methylation and histone modifications, together with deregulation of several microRNAs, is critical for establishing and stabilizing the activated phenotype of RASF [3,4]. The dynamic nature of epigenetic modifications, as described by Adrian Bird, ‘to register, signal or perpetuate altered activity states’ through ‘the structural adaptations of chromosomal regions’ [5] allows RASF to adapt their gene expression to the highly reactive microenvironment of the rheumatoid synovium. Furthermore, the inherent heritability of DNA and histone epigenetic marks through cell division ensures that, once activated, RASF remain permanently imprinted and thus independent of the inflammatory milieu, as shown *in vivo* in the severe-combined immunodeficiency mouse co-implanted with human cartilage and RASF [1,2].

Current disease modifying and anti-cytokine therapies, despite effectively halting or slowing inflammation and progression of RA, offer rather limited protection against ongoing joint destruction, with a substantial number of patients responding inadequately or not at all. Although these treatments can dampen the destructivity of RASF, reversing the activated phenotype of RASF remains unaccomplished; almost as a rule, arthritis flares up after discontinuing treatment and a cure for RA has not been found (yet).

Targeting RASF is key to developing joint-protective strategies in RA. Researchers are acutely aware of this, as exemplified by great interest in the American College of Rheumatology 2012 basic research conference on fibroblasts in rheumatic diseases. The extent to which epigenetic aberrations affect the activation of RASF, coupled with their dynamic nature and reversibility, makes them promising therapeutic targets, not only in cancer [6], but also in RA. Restoring the altered epigenetic patterns early in the pathogenesis of disease may prove effective in precluding the development of more chronic and aggressive disease. For example, we have shown that the promoter of a chemokine (C-X-C motif) ligand 12 (CXCL12) is hypomethylated in RASF, resulting in increased production of CXCL12 and CXCL12-dependent upregulation of matrix-degrading enzymes - matrix metalloproteinases (MMPs) [7]. Early structural changes in cartilage are required for the attachment and invasion of RASF [8] and cartilage damage seems necessary for RASF-mediated spreading of arthritis to unaffected joints [2]. Since RASF are the major synovial source of MMPs, it is imperative that restoring CpG methylation of the CXCL12 promoter by epigenetic treatments would decelerate the vicious cycle of progressive joint destruction in RA. Recently, several other aberrantly methylated genomic loci were identified in RASF genes regulating inflammation, extracellular matrix interaction, cell adhesion and migration [9], substantiating the role of defective DNA methylation in the pathogenesis of RA. Additionally, the RASF genome is globally hypomethylated [10]. As a consequence, the endogenous retroviral long interspersed element 1 is reactivated in RASF and can be detected in the synovial lining and at sites of cartilage invasion, the hot spots of active disease [3,10]. We have shown that a relative deficiency of DNA methyltransferase 1 in proliferating RASF, which can be further worsened by proinflammatory cytokines, contributes to the global loss of CpG methylation through cell division of RASF [10]. Furthermore, the consumption of S-adenosylmethionine, a principal methyl donor in DNA methylation, is significantly accelerated in RASF due to increased activity of spermidine/spermine N1-acetyltransferase and enhanced polyamine recycling [11]. Supplementing S-adenosylmethionine with a concomitant inhibition of spermidine/spermine N1-acetyltransferase may therefore represent a promising therapeutic approach in RA and is the first epigenetic strategy to directly act on RASF. As a proof of principle, targeting the globally deregulated DNA methylation has shown effective anti-cancer potential; namely, 5-azacytidine and 5-aza-2-deoxy-cytidine, both inhibitors of DNA methyltransferases, are clinically approved epigenetic drugs for treating high-risk myelodisplastic syndrome, counteracting DNA hypermethylation in cancer cells [6].

Beside deregulated DNA methylation, altered patterns of histone modifications can be found at the promoters of key genes in RASF, including secreted frizzled-related protein 1, which regulates Wnt signaling [12]. Furthermore, histone modifying enzymes, specifically the histone methyltransferase enhancer of zeste homolog 2 and histone deacetylases (HDACs), which are responsible for depositing and removing epigenetic marks, are misregulated in RASF and proinflammatory cytokines potentiate this misregulation [3,12]. Despite the complexity of histone acetylation, pan-inhibitors of HDACs (HDACi) have demonstrated impressive pre-clinical and clinical anti-cancer activities, reflected by their anti-proliferative, differentiation-inducing and pro-apoptotic effects in cancer cells, and are currently approved for the treatment of cutaneous T cell lymphoma [6]. HDACi have also consistently showed success in alleviating inflammation and preventing joint destruction, as prophylactic and therapeutic regimens, in several rodent models of arthritis [3]. Nevertheless, histone-modifying enzymes, including HDACs, can target several other non-histone proteins, such as transcription factors like nuclear factor kappa B (NF-κB), that are central to the pathogenesis of RA [3,13]. The anti-arthritic effects of HDACi should therefore be carefully interpreted in terms of restoring histone acetylation patterns in RASF. In this regard, several histone-unrelated anti-inflammatory mechanisms of HDACi have recently been demonstrated in RASF, including inhibition of nuclear retention of NF-κB and acceleration of interleukin-6 mRNA decay [13].

Deregulated microRNA networks add another level to the complexity of the activation of RASF. MicroRNA-203 is overexpressed in RASF, enhancing the secretion of MMP-1 and interleukin-6 [4]. Tumor necrosis factor-α-induced microRNA-18a activates RASF through a feedback loop in NF-κB signaling [14]. Furthermore, microRNA-155 plays a proinflammatory role in clinical and experimental arthritis, and microRNA-155-deficient mice are resistant to collagen-induced arthritis, suggesting microRNA-155 as an intriguing therapeutic target in RA [15].

## Conclusion

Targeting RASF by epigenetic agents provides a rationale for new therapeutic strategies in RA. Given the dynamic plasticity of the epigenome, the rapidly increasing knowledge in the field of epigenetics, and the recent implementation of epigenetic drugs in cancer treatment, epigenetic targeting of RASF appears to be within future reach.

## Note

This article is part of the collection ‘*Why is there persistent disease despite aggressive therapy of rheumatoid arthritis?*’, edited by Pierre Miossec. Other articles in this series can be found at http://arthritis-research.com/series/residual.

## Abbreviations

CXCL12: Chemokine (C-X-C motif) ligand 12; HDAC: Histone deacetylase; HDACi: Inhibitors of histone deacetylases; MMP: Matrix metalloproteinase; NF-κB: Nuclear factor kappa B; RA: Rheumatoid arthritis; RASF: Rheumatoid arthritis synovial fibroblasts.

## Competing interests

The authors declare that they have no competing interests.
